# Development and Characterization of Defect-Free Matrimid^®^ Mixed-Matrix Membranes Containing Activated Carbon Particles for Gas Separation

**DOI:** 10.3390/polym10010051

**Published:** 2018-01-08

**Authors:** Fynn Weigelt, Prokopios Georgopanos, Sergey Shishatskiy, Volkan Filiz, Torsten Brinkmann, Volker Abetz

**Affiliations:** 1 Helmholtz-Zentrum Geesthacht, Institute of Polymer Research, Max-Planck-Straße 1, 21502 Geesthacht, Germany; fynn.weigelt@hzg.de (F.W.); sergey.shishatskiy@hzg.de (S.S.); volkan.filiz@hzg.de (V.F.); torsten.brinkmann@hzg.de (T.B.); 2 Institute of Physical Chemistry, University of Hamburg, Martin-Luther-King-Platz 6, 20146 Hamburg, Germany

**Keywords:** mixed-matrix membranes, Matrimid^®^, activated carbon, time-lag, permeability, gas separation

## Abstract

In this work, mixed-matrix membranes (MMMs) for gas separation in the form of thick films were prepared via the combination of the polymer Matrimid^®^ 5218 and activated carbons (AC). The AC particles had a mean particle size of 1.5 μm and a mean pore diameter of 1.9 nm. The films were prepared by slow solvent evaporation from casting solutions in chloroform, which had a varying polymer–AC ratio. It was possible to produce stable films with up to a content of 50 vol % of AC. Thorough characterization experiments were accomplished via differential scanning calorimetry and thermogravimetric analysis, while the morphology of the MMMs was also investigated via scanning electron microscopy. The gas transport properties were revealed by employing time-lag measurements for different pure gases as well as sorption balance experiments for the filler particles. It was found that defect free Matrimid^®^ MMMs with AC were prepared and the increase of the filler content led to a higher effective permeability for different gases. The single gas selectivity *α_ij_* of different gas pairs maintained stable values with the increase of AC content, regardless of the steep increase in the effective permeability of the pure gases. Estimation of the solubilities and the diffusivities of the Matrimid^®^, AC, and MMMs allowed for the explanation of the increasing permeabilities of the MMMs, with the increase of AC content by modelling.

## 1. Introduction

Membranes offer attractive opportunities for many gas separation applications, e.g., in hydrogen recovery, natural gas processing, etc. [1]. In comparison to conventional gas separation processes like adsorption, absorption, or condensation, membrane technology offers an easier separation process. For membranes, polymers allowing for mass transfer according to the solution–diffusion mechanism are often used [1]. The benefits of using polymers are that they have a good processability and a low price, whereas they also have a low stability at high temperatures and aggressive chemical compounds. Robeson compiled in his work the separation capacity for polymeric membranes for the gas pairs consisting of He, H_2_, O_2_, N_2_, CO_2_, and CH_4_. As a result, it was shown that the maximal selectivity inversely correlates with the permeability: with increasing the permeability, the selectivity decreases. This upper limit is called “Robeson Upper Bound” and was first established in 1991 [2] and updated in 2008 [3]. In contrast to polymers, inorganic membranes made of zeolites or carbon exhibit high permeabilities and selectivities [4,5]. Nevertheless, the disadvantage for industrial use complies that they are often mechanically and chemically very unstable and cost-intensive. Because of this, an alternative approach was proposed, in which inorganic particles are dispersed in the polymeric matrix to combine the positive properties of the two material classes. This kind of membranes is often called mixed-matrix membranes (MMMs) [6,7], which are based in most of the cases on a polymer matrix and inorganic fillers (porous or non-porous). These components are optimally combined to exhibit enhanced separation and mechanical properties in comparison to the pure materials. In principle, almost all polymers can be used for the fabrication of MMMs, as far as they can be easily processed, e.g., in solution. Usually, the solution method is favored for the dispersion of the inorganic fillers since the polymers are relaxed in the solution and the inorganic fillers can be easily incorporated. Removing the solvent in a proper way results in the formation of the MMMs. Numerous combinations of polymers and fillers have already been studied and extensive reviews have been published [8,9,10]. Elastomers, as well as semi-crystalline polymers (e.g., polydimethylsiloxane polyethylene respectively), have been used for such applications. Also, a variety of filler materials have been studied, among them, metal–organic frameworks (MOFs), polyoctahedral silsesquioxanes, zeolites, carbon nanotubes, activated carbon, as well as ceramic, metal oxide particles (e.g., γ-alumina, titanium dioxide nanoparticles) and metallic nanoparticles, e.g., palladium [7,11,12,13,14,15,16,17,18].

Polyimides are a class of polymers that are prepared via polycondensation reaction between a dianhydride and a diamine or diisocyanate [19], and they usually exhibit high glass transition temperatures. Matrimid^®^ 5218 (further Matrimid^®^) belongs to this category of polymers and is well-known for its attractive gas separation properties, in particular for a good gas separation performance in the separation of CO_2_ from CH_4_ or for hydrogen recovery [20]. There is a variety of works on Matrimid^®^ MMMs. Vinoba et al. give a good overview on recent fillers used in MMMs [9], while Wang et al. recently gave an overview of the use of mixed matrix membranes for CO_2_ separation applications [10]. Khan et al. published a work on Matrimid^®^ MMMs with silica particles exhibiting a decreasing trend for the activation energy of permeation of CO_2_ with increasing silica particles content [21]. Naseri et al. reported the fabrication of Matrimid^®^ MMMs with metal–organic frameworks (MOFs) and found that the selectivity of the composite material was improved for the pair of gases CO_2_/CH_4_ and CO_2_/N_2_ in comparison to the pure Matrimid^®^ for a content of MOFs equal to 30 wt % [22]. Similar are the results published by Dong et al. with the use of a different MOF. In this case, they observed that the selectivity for CO_2_ increases but they also studied the morphology of the membranes and found a “crater” structure of the Matrimid^®^ matrix phase around the MOF particles. In this work, the thermal stability of Matrimid^®^ was also investigated and found to be better since the glass transition temperature showed a significant increase due to the introduction of the inorganic particles [23]. Last but not least, significant results were reported by Zhang et al. on MMMs with mesoporous ZSM-5 zeolite particles. In this case, the micropores of the ZSM-5 crystals provided size and shape selectivity especially for the gas pairs of H_2_/N_2_ and O_2_/N_2_ [24].

Activated carbons (AC) are materials also well known for gas separation processes. Recently, our group published works on MMMs prepared as thin film composite membranes based on polydimethylsiloxane (PDMS) or polyoctomethylsiloxane (POMS) with AC. With the use of AC, it was possible to increase the separation performance towards higher hydrocarbons. The selectivity for a binary mixture of *n*-C_4_H_10_/CH_4_ was increased [25,26].

In this work, we present the fabrication of MMMs based on Matrimid^®^ and AC. The target of this work is to identify the properties of the prepared MMMs not only regarding the gas transport performance but also concerning the material characteristics. The analysis was done via a variety of experiments, which are presented below. The correlation of the material properties, as well as the gas transport properties, e.g., the morphology and the thermal properties, with the permeability, revealed interesting characteristics for the overall separation performance. 

## 2. Materials and Methods

### 2.1. Materials

The Matrimid^®^ 5218 (Matrimid^®^), a polyimide of 3,3′,4,4′-benzophenone tetracarboxylic dianhydride and diamino-phenylindane, was purchased from Huntsman Advanced Materials GmbH (Bergkamen, Germany) in powder form and it was used as the matrix for the mixed matrix membranes. The bulk density of Matrimid^®^ is 1.24 g cm^−3^.

The activated carbon (AC) was used as inorganic filler and was kindly provided by the company Blücher GmbH (Erkrath, Germany). The AC particles were produced from a polymeric precursor that leads to clean carbon particles with reproducible pore structure. The initially prepared spherical particles of 100 μm diameter were milled and fractioned to a final mean particle size *d*_50_ = 1.5 μm without changes of the pore characteristics according to the producer. The apparent density of the particles was 0.89 g cm^−3^ and the mean pore size was 1.87 nm [25].

The solvent and non-solvent used for Matrimid^®^ membrane production were chloroform (analytical grade, Merck GmbH, Darmstadt, Germany) and methanol (analytical grade, Merck GmbH, Darmstadt, Germany), respectively. 

### 2.2. Preparation of Membranes

Thick films of Matrimid^®^ and MMMs of Matrimid^®^ and AC were fabricated by solution casting using chloroform as a solvent. Solutions with different contents of AC were prepared while maintaining the same amount of polymer. The polymer solution prepared had a concentration 4 wt %. The films produced by the solution casting had an activated carbon content of 0–50 vol %. The typical procedure to prepare the films was as follows: first, the dried powder of Matrimid^®^ together with the activated carbon particles were dissolved—dispersed in chloroform (CH_3_Cl) and stirred for two hours. Following that, the solution was ultrasonicated using an Elmasonic ultrasonication device (Elma Schmidbauer GmbH, Singen, Germany), which did not affect the molecular weight of the polymer. In the next step, the solution was directly cast into a leveled Teflon^®^ mold and solvent evaporation was carried out with a constant nitrogen flow under ambient conditions. The dried membranes were processed with methanol for 8 h to remove the residues of chloroform via solvent exchange [27]. In the last step, the membranes were dried at 60 °C for 24 h under vacuum in order to completely remove solvent residues. The thickness of the obtained membranes was in the range of 80 up to 160 μm, dependent on the amount of AC. The thickness was measured using a Deltascope FMP10 (Helmut Fischer GmbH, Sindelfingen, Germany), in order to obtain accurate values. 

### 2.3. Characterization Methods

#### 2.3.1. Thermal Analysis

*Differential Scanning Calorimetry*. Differential scanning calorimetry (DSC) experiments were carried out using the calorimeter DSC 1 (Mettler-Toledo, Gießen, Germany), in a nitrogen atmosphere, with a heating rate of 10 K/min, within a temperature range from room temperature to 380 °C. Three heating–cooling cycles were accomplished with a five minutes isotherm interval between the heating and the cooling. The first heating interval served for erasing the sample thermal history from the preparation and started from room temperature up to 380 °C, while the two other cycles were used for the determination of thermal properties and they were accomplished in the temperature range from 200 °C up to 380 °C. The second heating interval was used for the evaluation of the glass transition and the third heating interval was used to verify it. The glass transition temperature *T*_g_ was assessed as the inflection point of the heat flow as a function of the temperature with the onset method using the instrumentation software. The DIN midpoint was calculated with the use of the software of the instrumentation. Approximately 10 mg of the polymer and the grounded composites were placed in an aluminum pan of 10 μL after having been dried for several days under vacuum. 

*Thermogravimetric Analysis*. Thermogravimetric analysis (TGA) was performed using a TG 209 F1 Iris (Netzsch, Selb, Germany). The experiments were carried out within a temperature range from 25 °C up to 1000 °C at a heating rate of 10 K/min. The measurements were performed under argon atmosphere. Approximately 10 mg of the membrane was placed in a ceramic pan for the measurements.

#### 2.3.2. Morphological Characterization

*Scanning Electron Microscopy (SEM).* The morphology of the membranes was investigated with the scanning electron microscope Merlin (Carl ZEISS GmbH, Oberkochen, Germany). The samples were cryo-fractured in liquid nitrogen to examine the dispersion of the filler particles in the cross-section. The surface morphology was investigated as well. The samples were sputter-coated with approximately 2 nm Pt. The images were obtained at an acceleration voltage in the range of 3–5 kV. 

#### 2.3.3. Gas Sorption Measurements

The sorption of pure gases He, H_2_, O_2_, N_2_, CO_2_, and CH_4_ on AC was accomplished with the gravimetric sorption analyzer IsoSORP^®^ Static (Rubotherm GmbH, Bochum, Germany). The system is equipped with a precision pressure sensor DPI 282 of ±0.006 bar accuracy. The experimental temperature was maintained with a cryo-compact circulator (Julabo GmbH, Seelbach, Germany) with an accuracy of ±0.03 °C. Adsorption isotherms were recorded at 30 °C in a pressure range from ~0 up to 30 bar. The resolution and reproducibility of the magnetic suspension balance (MSB, Rubotherm GmbH, Bochum, Germany) was 0.01 mg and ±0.03 mg, respectively. In order to correlate with the real gas behavior, the fugacities of the different gases were determined according to the Soave–Redlich–Kwong equation of state [28] using the chemical process simulation and optimization software Aspen Plus^®^ (AspenTech, Bedford, MA, USA).

#### 2.3.4. Gas Transport Characteristics Determination

**Figure 1:**
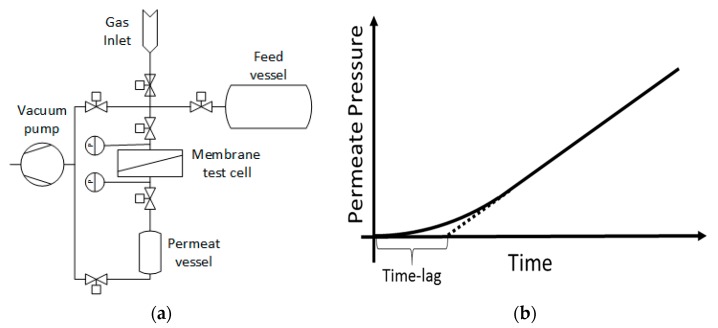
(**a**) Sketch of the time-lag measurement setup and (**b**) the schematic representative of the experimental result (solid curve: permeate pressure; dotted line: tangent at large time).

The gas transport parameters were determined with the time-lag (variable pressure, constant volume [29]) method at 30 °C and 1000 mbar feed pressure for He, H_2_, N_2_, O_2_, CO_2_, and CH_4_ [30]. The method relies on maintaining a constant feed pressure and measuring the permeate pressure changing as a function of time due to the diffusion of gas molecules through the membrane film of known thickness. The membrane under investigation was placed into the measurement cell and sealed with a Viton^®^ O-ring, which served as a barrier between the feed and permeate side of the measurement instrumentation. Prior to the measurement, the apparatus was evacuated to a state where no evidence of desorption was observed anymore, i.e., no pressure increase was recorded. The pressure increase in the permeate chamber with known constant volume was monitored from the moment the gas at a constant pressure was brought in contact with the membrane. From the obtained curve, the time-lag, *θ*, was determined by extrapolating the slope of the linear increase to its intersection with the time axis and the gas permeability coefficient of the membrane was calculated from the linear part of the curve [31]. The employed experimental apparatus as well as the schematic representation of the experimental result is depicted in Figure 1. The diffusion coefficient of component *i* (*D_eff,i_*) was calculated by the following equation [32]:

(1) $$\theta =\frac{l^{2}}{6D_{eff,i}}$$

where *l* is the membrane film thickness and *D_eff_* the effective diffusion coefficient of the penetrant in the studied material. The permeability (*P_eff,i_*) of a gas penetrant *i* is the pressure or fugacity difference (i.e., driving force) and thickness-normalized flux of the component through the membrane and is defined by:

(2) $$P_{eff,i}=\frac{N_{i}l}{A_{m}\Delta p_{i}}$$

where *P_i_* is the permeability of component *i*, *N_i_* is the component *i*’s volumetric flowrate at standard conditions (STP) through the membrane, *A_m_* is the membrane area, and Δ*p_i_* the partial pressure (or fugacity) difference between feed and permeate sides. The volumetric flowrate was determined from the permeate pressure change with time assuming that in a pressure range 0–10 mbar the ideal gas law is applicable. The solubility (*S_eff,i_*) coefficient can be calculated from the following equation:

(3) $$P_{eff,i}=D_{eff,i}S_{eff,i}.$$

The selectivity of a dense gas separation membrane is defined as:

(4) $$\alpha_{ij}=\frac{P_{i}}{P_{j}}=\left(\frac{S_{i}}{S_{j}}\right)\left(\frac{D_{i}}{D_{j}}\right).$$

Here *P_i_* and *P_j_* are the permeabilities of gas *i* and *j*.

For the time-lag measurements, a custom-made machine was used. The gases used for the experiments (Linde AG, Munich, Germany) were of high purity, while a thermostat, similar to the one employed in the sorption experiments, was used to maintain the temperature. The vacuum was generated by a turbomolecular pump (Pfeiffer GmbH, Asslar, Germany). A LabView based custom software (National Instruments, Austin, TX, USA) was used for the control of the time-lag experiments as well as for the evaluation of the data. It should be noted that the placement of the Viton^®^ O-ring involves relatively hard pressing of the O-ring onto the membrane. The O-ring has a 65 Shore hardness. The pressing ensures a good sealing of the membrane in the measurement cell, whilst it is also an indication of the membrane mechanical stability, which is necessary for the accomplishment of the measurement.

## 3. Results and Discussion

### 3.1. Thermal Analysis

**Figure 2:**
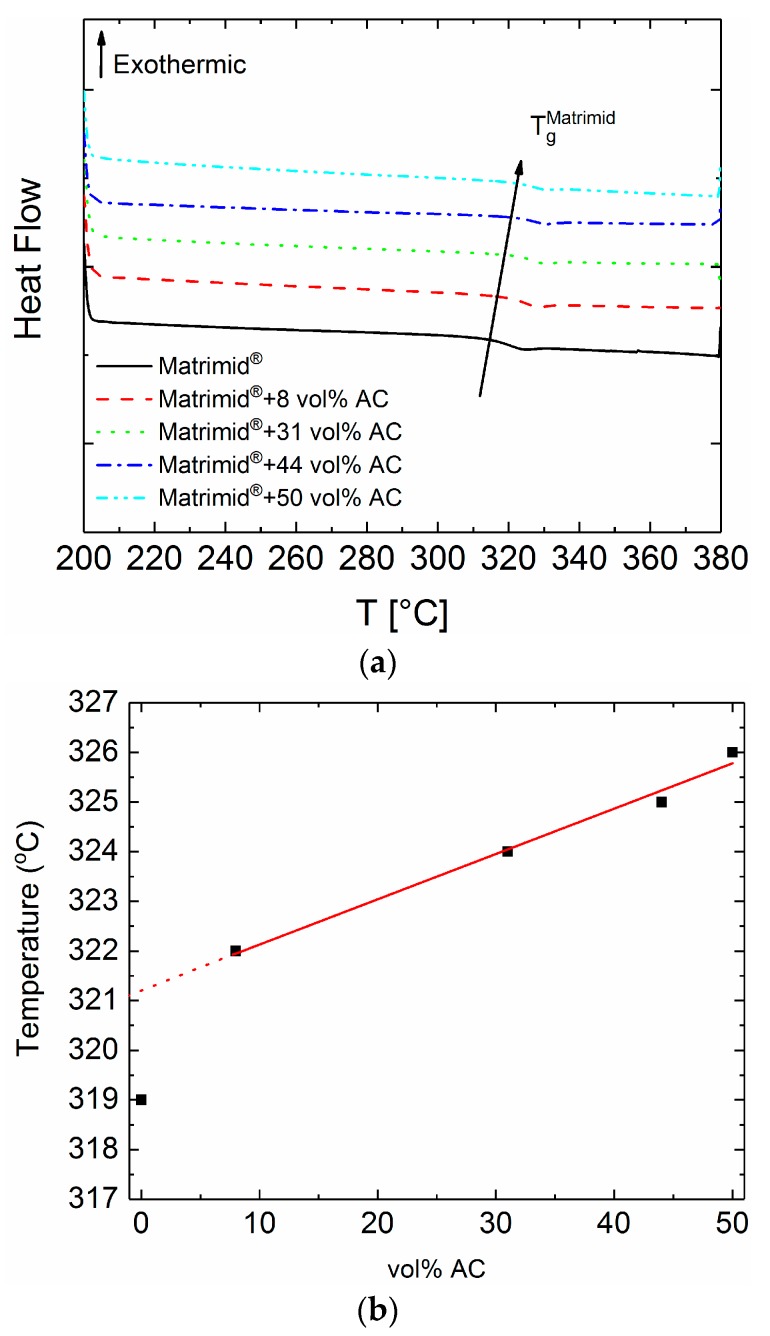
(**a**) DSC thermographs for the pure Matrimid^®^ and the Matrimid^®^–activated carbon (AC) mixed-matrix members (MMMs). The data are vertically shifted for clarity. (**b**) Glass transition as a function of the AC volume content. The linear fit for the MMMs indicates the slight linear increase of the glass transition due to the well adaption of the AC particles in the polymer matrix.

*Differential Scanning Calorimetry.* The differential scanning calorimetry of the pure Matrimid^®^ and the Matrimid^®^–AC MMMs shows a small difference between the glass transition temperature of the pure polymer and the MMMs. More in detail, as it can be seen from the values in Table 1, the glass transition temperature is slightly but gradually affected by the incorporation of AC into Matrimid^®^. This indicates that there is some interaction of the polymer with the AC [33,34]. The pure Matrimid^®^ shows a glass transition temperature at 319 °C and it is increasing with the increase of activated carbon content up to the value of 326 °C for the MMM with 50 vol % filler content. The slight interaction of the filler particles with the polymer matrix was expected, since the carbon particles are not chemically modified (e.g., containing functional groups, hydrophobically or hydrophilically modified) and therefore a strong bonding of the polymer matrix with the particles is not to be expected. The results are presented in Figure 2a. In other cases, incorporation of chemically modified fillers have shown a significant effect on the glass transition temperature of Matrimid^®^, attributed to the chemical bonding—hydrogen bonds mostly—occurring at the interface between the polymer and the filler particles [24]. It is important to mention that the increase of the glass transition with the increase of the AC volume content (Figure 2b) is linear for the MMMs, but the extrapolation of the linear fit to 0 vol % AC indicates a difference. A slight increase of 2 °C for the theoretically expected glass transition temperature for the pure Matrimid^®^ (extrapolated value is 321 °C), in comparison to the experimentally estimated value of 319 °C, is observed. This indicates that the polymer incorporates well the particles into its matrix, as it is also pointed out in literature [33,34]. 

**Figure 3:**
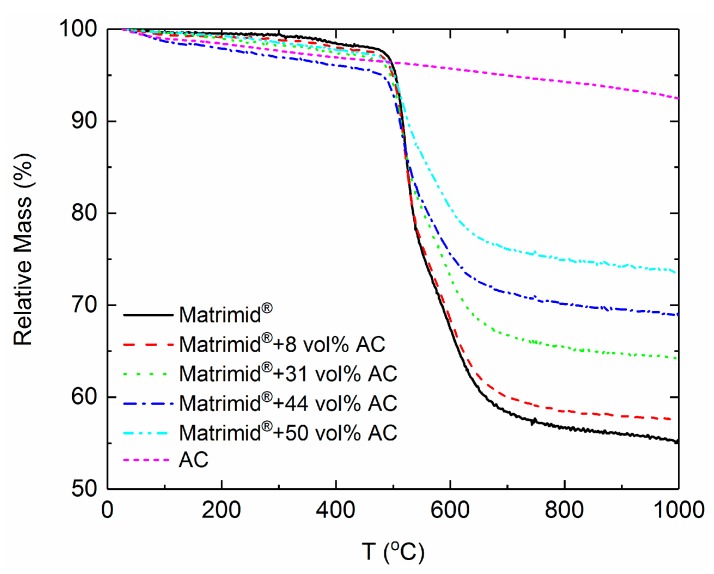
TGA thermographs of the MMMs prepared in this work. The pure Matrimid^®^ is also shown for comparison.

*Thermogravimetric analysis*. In Figure 3, the results of the TGA are presented for the pure Matrimid^®^ as well as the Matrimid^®^–AC MMM prepared in this work. It is indicated that the membranes show a high thermal stability since the degradation processes start above 400 °C. The initial mass-losses indicate changes of the polyimide polymer as well as small changes of the AC.

From the TGA curves, it is observed that the AC presence in the MMMs does not significantly influence the onset of the Matrimid^®^ decomposition. As it follows from the polymer decomposition curve, the process of polymer decomposition does not occur in one-step and the shape of the curve changes when the AC is present in the polymer. The remaining weight of the sample at 1000 °C was used to determine the real weight content of the AC in the Matrimid^®^ MMMs according to the following Equation (5):

(5) $$w_{AC}=\frac{m_{r,MMM}-m_{r,Matrimid}}{m_{r,AC}-m_{r,Matrimid}}\times 100$$

where *m_r,i_* is the remaining relative mass of Matrimid^®^, AC, and MMMs, and *w_AC_* is the weight content of AC in the MMMs. In order to calculate the volume content $\varphi_{AC}$ of the AC in the films, the densities of the pure materials are needed. The calculation is done following the Equation (6):

(6) $$\varphi_{AC}=\frac{1}{1+\left(\frac{\rho_{AC}}{\rho_{Polymer}}\right)\cdot \left(\frac{1}{w_{AC}}-1\right)}$$

where *ρ_Polymer_* is the density of Matrimid^®^ and *ρ_AC_* is the density of AC. In Table 2, the theoretical and the estimated values of AC content from the TGA are given for all the MMMs studied in this work. The small deviation from the initial content of the AC that was used for the membrane fabrication is caused by the sample preparation.

### 3.2. Morphology of the Membranes

**Figure 4:**
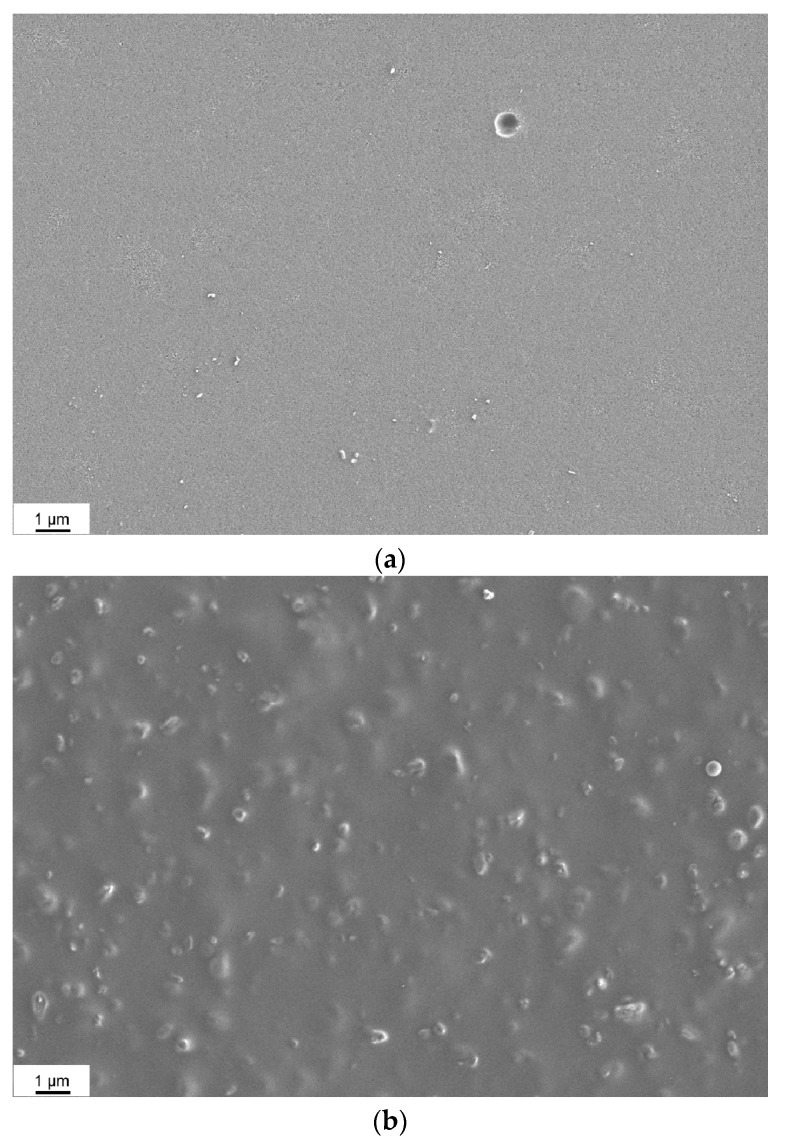
SEM images of (**a**) the surface of a pure Matrimid^®^ membrane; (**b**) surface of a Matrimid^®^ MMM with 44 vol % AC.

**Figure 5:**
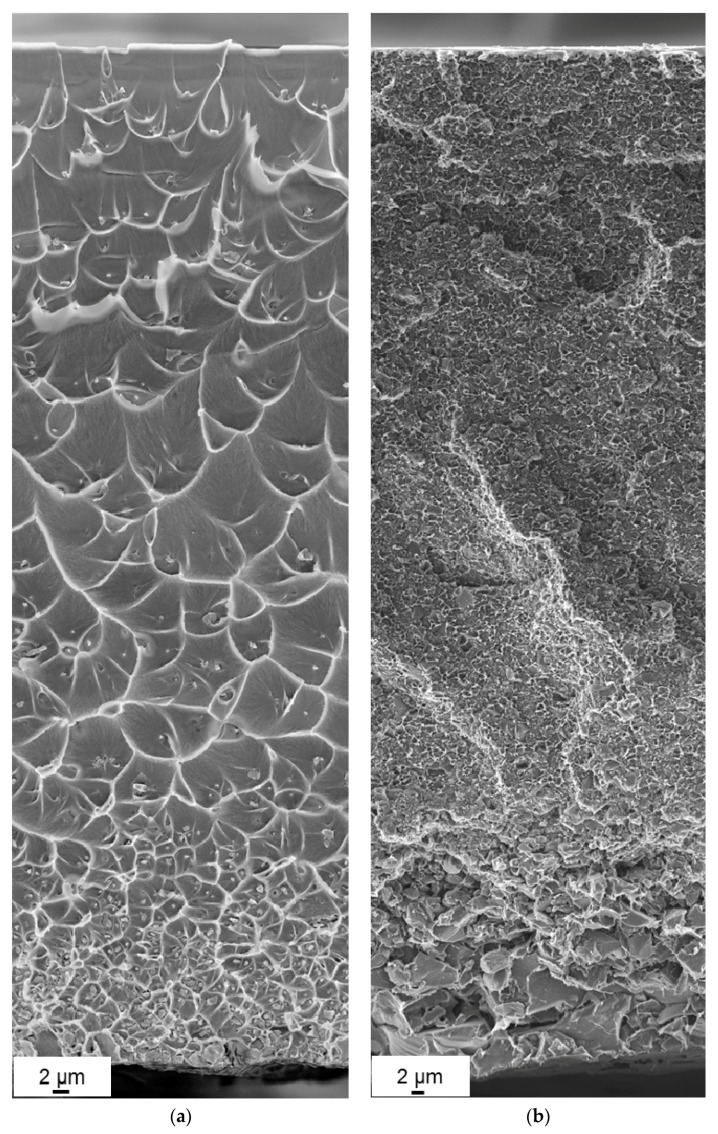
SEM image of a fracture surface of a Matrimid^®^ MMM with (**a**) 8 vol % AC and (**b**) 50 vol % AC. The increased concentration of the AC particles at the bottom of the membrane due to the membrane preparation is visible. The “crater”-like morphology is observed in the case of the 8 vol % AC while it is not visible for the 50 vol % AC due to the high content of fillers.

Surface and cross-section morphologies for the MMMs of Matrimid^®^ and AC have been analyzed. In Figure 4a,b, representative SEM surface images of the pure Matrimid^®^ and the filled MMMs are shown. The topography is smooth in case of the pure Matrimid^®^ membrane while due to the incorporation of the AC in the MMMs the roughness is increased. The surface of the membranes does not exhibit defects that could affect the gas separation properties. The filler particles are covered by a thin layer of polymer, which indicates the good compatibility of these different materials as it was already concluded from the thermal analysis. Additional information was revealed from the cross-section images. In Figure 5, it is shown that the AC particles are well incorporated into the polymer matrix as already indicated by the surface view of Figure 4b. A continuous solid interface between the carbon particles and the polymer was found from the SEM. This is in agreement with literature [23,24]. 

Figure 5a of the MMM with 8 vol % of AC shows an interesting type of morphology of the composite is indicated. This morphology is the “crater” like morphology deriving from the inclusion of the activated carbon into the polymer matrix, which is also referred to in literature [22,35,36]. Also, the ductile behavior during fracture of the Matrimid^®^ MMM is visible. The particles act as the centers of the ductile fracture and this is also in agreement with literature [37]. Furthermore, the morphology is affected strongly by the incorporation of a high amount of filler particles. As can be seen in Figure 5b of the MMM with 50 vol %, the polymer domains that include the filler particles are now significantly smaller and almost not detectable. A closer observation of the bottom part of Figure 5a—bottom part of the cast membrane with 8 vol % AC particles—indicates an increase of particle concentration in that area, similar to what is also mentioned by Fernández-Barquín et al. in case of mixed-matrix membranes of zeolite with poly(1-trimethylsilyl-1-propyne) (PTMSP) [38]. This can be explained by the method of the membrane fabrication through solvent evaporation. The solvent does not evaporate instantly; rather, the controlled solvent evaporation is effected over a period of more than 24 h by applying a small nitrogen flowrate through the covering compartment above the membrane preparation mold. This gives the solvent enough time to penetrate into the small pores of the filler particles. In that way, the particles are filled up with chloroform and obtain higher density so that they partially sediment. Nevertheless, since the polymer and the particles are well compatible, the polymer keeps the particles in place and the membrane is absolutely stable, allowing for easy handling. Another indication of the good stability is that the membranes were not raptured during the gas transport measurement. Hence, there is a prospect of further AC modification by incorporation of substances able to interact specifically with components of the gas mixture to be separated into the porous structure and thus increase membrane performance.

### 3.3. Gas Transport Characterization Results

**Figure 6:**
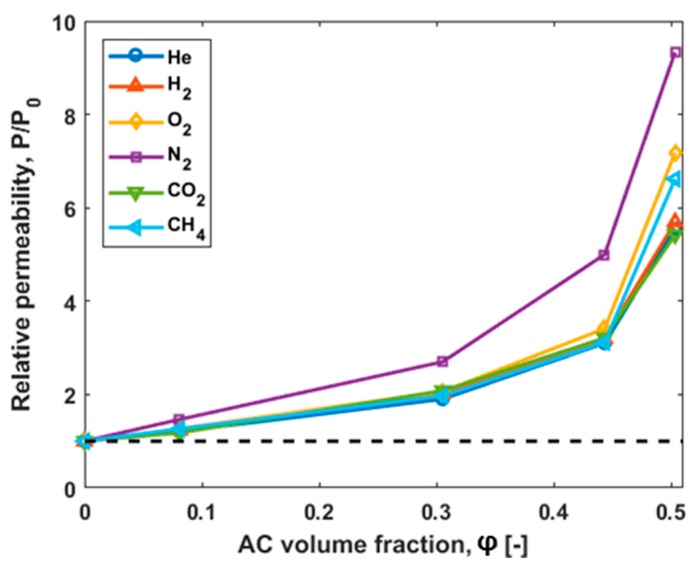
Ratio of permeability of MMMs (P) to that of unfilled polymer (P_0_) as a function of the AC volume fraction. The relative enhancement of permeability of MMMs as a function of AC volume fraction at 30 °C depends on the gas (dotted line: relative permeability for Matrimid^®^).

The time-lag method was used for the evaluation of the permeability, the diffusivity and the solubility coefficients of the MMMs for the gases He, H_2_, O_2_, N_2_, CO_2_, and CH_4_. The pure gas permeability data determined for Matrimid^®^ and the MMMs at 30 °C and 1000 mbar feed pressure are presented in Table 3. The gas permeabilities of filled Matrimid^®^ are higher than those of pure Matrimid^®^ and increase as the filler volume fraction increases. The trend for all gases is shown in Figure 6, which presents the relative permeability of Matrimid^®^ compared to the filled Matrimid^®^ MMMs as a function of the volume fraction. 

**Figure 7:**
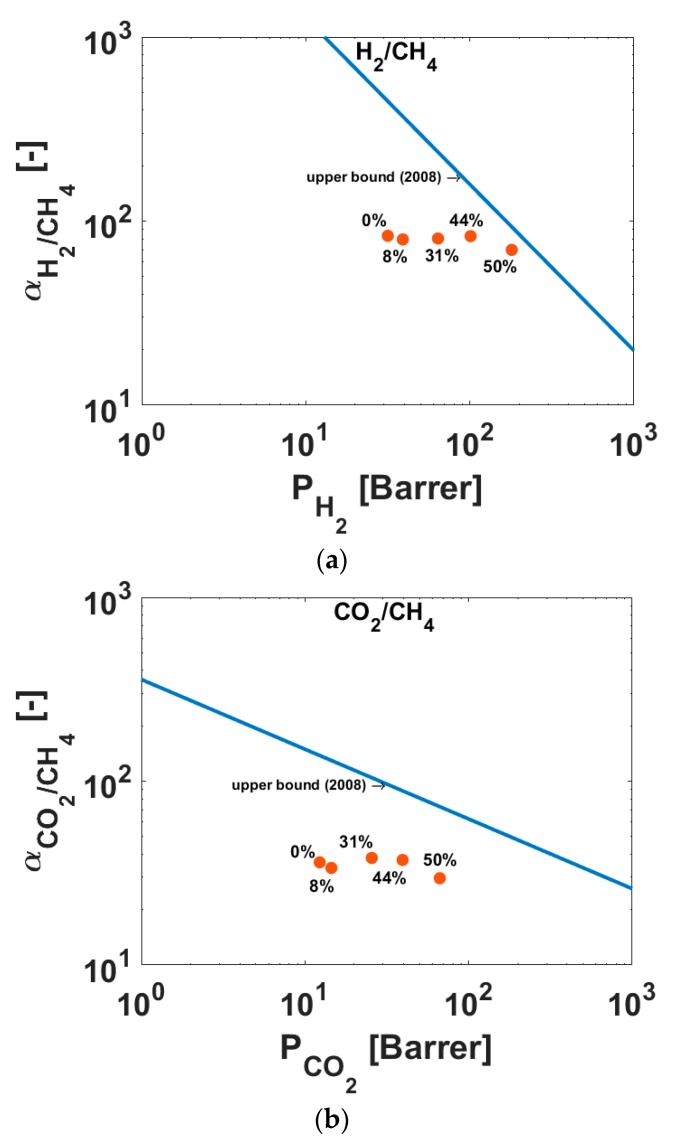

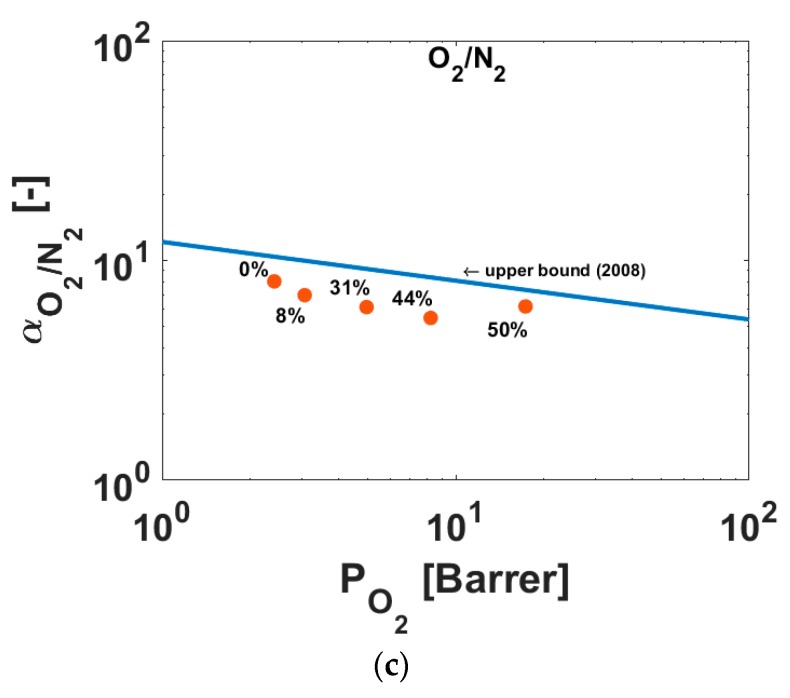
(**a**) H_2_/CH_4_, (**b**) CO_2_/CH_4_ and (**c**) O_2_/N_2_ permselectivities of MMMs for different loadings of AC particles at 30 °C.

With an AC content of 50 vol %, strongly increasing permeabilities for all gases are observed. For example, the permeability coefficient of hydrogen changes from 31.6 Barrer in case of the pure Matrimid^®^ to 180 Barrer for the MMM with 50 vol % AC content; here the permeability increases by a factor of 5.7. The permeability of CH_4_ increases from 0.34 Barrer (pure Matrimid^®^) to 2.25 Barrer (50 vol % AC in Matrimid^®^), meaning that the permeability increases with a factor of 6.6. This means that a slight change in the permselectivities of H_2_/CH_4_ occurs. In Figure 7a, the permselectivities of H_2_/CH_4_ over the permeabilities of H_2_ are shown for the different membranes. In the same plot, the Robeson upper bound (2008) [3] is presented and it is observed that the performance of the produced membranes approaches this bound with increasing amounts of activated carbon in the MMMs compared to pure Matrimid^®^. 

In Figure 6, the increase of the permeabilities of the MMMs compared to the permeability of the pure Matrimid^®^ is presented. For the gases CO_2_, H_2_, and He, the same increasing trend of the permeabilities was found. The permeabilities of CH_4_ and O_2_ increase slightly more while N_2_ has the highest increase. 

For CO_2_/CH_4_, a slight decrease of permselectivity over the amount of fillers is observed, due to the higher increase of CH_4_ permeability, but both permeabilities rise with the amount of filler. In Figure 7b the permselectivity of the gas pair CO_2_/CH_4_ is shown. Even though the permselectivity is decreasing, a closer approach to the Robeson bound (2008) with a higher amount of AC in the MMMs compared to pure Matrimid^®^ is observed, because of the highly increasing permeabilities of the gases. In Figure 7c, the permselectivity of O_2_/N_2_ over the permeability of O_2_ is shown and the same trend is observed. For the gas pairs H_2_/CH_4_, CO_2_/CH_4_, and O_2_/N_2_, the permselectivities are listed in Table 4.

Furthermore, in order to investigate the existence of defects on the membranes, the Knudsen selectivity was calculated employing Equation (7):

(7) $$\alpha_{i,j}^{Kn}=\sqrt{\frac{M_{j}}{M_{i}}}$$

where *M* is the molecular mass of the gas.

Comparing the permselectivities values with the calculated Knudsen selectivity values (Table 4) indicates that the values are not in agreement. Hence, the gas transport in the membranes is still controlled by the solution–diffusion mechanism of the polymer and the contribution of the dispersed AC particles. The trend of the increase of the permeabilities in the MMMs is based on the solubilities and diffusivities (see Equation (3)), and it will be explained further below. 

### 3.4. Sorption Results

The pure gas solubility coefficients of the investigated gases in Matrimid^®^ 5218 and the MMMs determined according to Equation (3) from time-lag experiments at 30 °C are presented in Table 5. 

**Figure 8:**
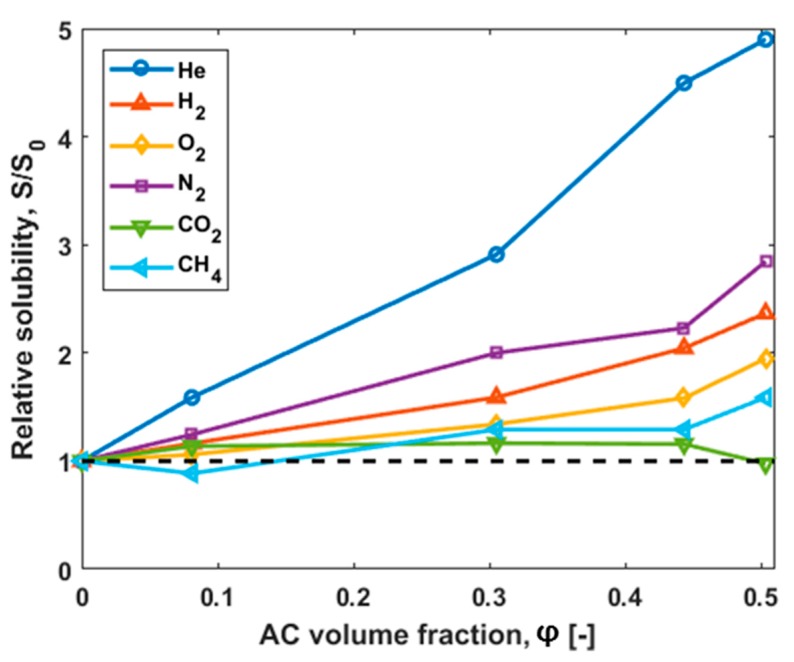
Ratio of solubility of MMMs (S) to that of unfilled polymer (S_0_) as a function of the AC volume fraction. The relative enhancement of solubility of gases in MMMs as a function of AC volume fraction at 30 °C depends on the gas (dotted line: relative permeability of Matrimid^®^).

The increasing trend of the relative solubility of the investigated gases in the MMMs with the increase of the AC content compared to the pure Matrimid^®^ is shown in Figure 8. 

To explain the trend for the sorption coefficient *S_eff_* in the MMMs, one should consider the adsorption isotherms of the activated carbon, which are crucial for the separation performance of the resulting MMMs. Adsorption isotherms of He, H_2_, O_2_, N_2_, CO_2_, and CH_4_ have been measured at 30 °C. The equilibrium can be described by the Langmuir-isotherm Equation (8) [39]. The equation for the isotherm is given as:

(8) $$q=q_{max}\cdot \frac{b\cdot p}{1+\left(b\cdot p\right)}$$

where *q* is the adsorbate concentration, *p* the pressure, and *q_max_* the maximum adsorbate concentration possible for a monolayer coverage of the considered component on the investigated adsorbent. The parameter *b* also is specific for an adsorbate–adsorbent pair.

**Figure 9:**
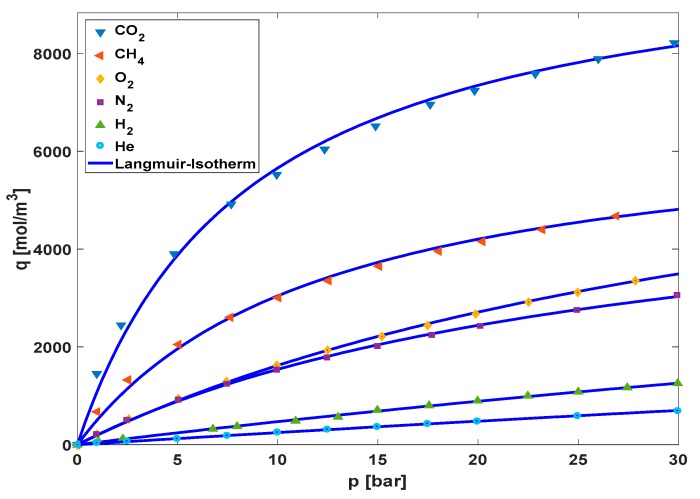
Experimentally measured concentrations for the different gases over the pressure and the corresponding adsorption-isotherm at 30 °C.

In Figure 9, the adsorption isotherms for the different gases are shown, and in Table 6 the Langmuir parameters are listed. 

As expected for AC adsorbents, CO_2_ has the highest maximum loading combined with the fastest kinetics, followed by methane, as an example for a light hydrocarbon gas. The permanent gases helium, hydrogen, oxygen, and nitrogen all show considerably smaller adsorption tendency. The difference between oxygen and nitrogen appears to be governed by the high maximum loading of oxygen. In contrast, the favorable adsorption of hydrogen in the investigated pressure range is assumed to be due to the faster kinetics, when compared to helium. 

The time lag experiments were done at low feed pressure (1000 mbar), which means that a Henry coefficient for the gas adsorption-isotherm can be obtained. At zero loading the Langmuir-isotherm Equation (8) simplified to the Henry Equation (9) [39].

(9) $$q=(q_{max}b)\cdot p=H\cdot p$$

where the Henry constant *H* is *q_max_b*.

In the most simple form, the adsorption in glassy polymers can also be assumed to show linear behavior (Equation (10)) [1],

(10) c=S·p

where *S* is the solubility coefficient of the pure Matrimid^®^, *c* the concentration which is adsorbed and *p* the pressure. 

The comparison of the two adsorption coefficients for the pure materials is possible. In Table 7, the Henry and the solubility coefficients for all gases are shown. The ratio between *H* and *S* follows the same trend as for the time-lag measurements. In more detail, the *H*/*S* ratio for CO_2_ is the smallest, and for He the ratio is the highest. As a result, the trend of the ratio of the pure materials follows the trend observed from the time-lag measurements (Figure 8) described in the following series:

CO_2_ < CH_4_ < O_2_ < H_2_ < N_2_ < He

### 3.5. Diffusion Coefficient

Analyzing the diffusion coefficients presented in Table 8 for all the studied membranes allows an estimation of the influence of the AC on the gas transport properties.

The AC has pores below 2 nm, meaning that the samples belong to the region of microporous materials and below the region of Knudsen diffusion [39]. Also, the gas component diameter is not much bigger than the AC pore diameter, leading to the conclusion that the AC behaves as a material where configurational diffusion is the dominant transport mechanism, i.e., the diffusion coefficient can be described by surface diffusion [40].

Since it was not possible to measure the diffusion coefficient of the AC directly, due to the feature of the powder and the experimental set up used, the diffusion coefficient was estimated from the time-lag measurements of the Matrimid^®^ MMMs. To calculate the diffusion coefficient for the AC, the values of pure Matrimid^®^ and the MMMs with 50 vol % are compared. The assumption is that the diffusion coefficient of AC has an influence of 50% in the MMMs with 50 vol % AC inside. This leads to the values of Table 9 for the diffusion coefficient of AC.

With Equation (11), it is possible to get a permeability for the inorganic filler for small pressures, since H_AC_ is only valid for small pressures.

(11) $$P_{AC,i}=D_{AC,i}\cdot S_{AC,i}$$

### 3.6. Permeability Predictions

To predict the permeability of MMMs, a number of models have been suggested. Shimekit et al. as well as Vinh-Thang and Laliagiune give an overview of existing models and by using experimental data they predict the permeability of MMMs for different gases [41,42]. The simplest model to be applied is the Maxwell model. The Maxwell model was originally developed to simulate the electrical conductivity of composite materials [43]. It was shown that the equation is also valid for the flux through membranes containing a dispersed phase in the matrix of a polymer [44]. The Maxwell model provides values valid only for low particle loadings (0 < *φ_d_* < 0.2). The description of the Maxwell model is given by the following Equation (12):

(12) $$P_{eff}=P_{c}\frac{P_{d}+2P_{c}-2\varphi_{d}\left(P_{c}-P_{d}\right)}{P_{d}+2P_{c}+\varphi_{d}\left(P_{c}-P_{d}\right)}$$

where *P_eff_* is the effective permeability, *P_c_* is the permeability of the continuous phase (Matrimid^®^), *P_d_* the permeability of the dispersed phase (AC), and *φ_d_* the volume fraction of the disperse phase (AC).

For medium particle loadings, the Bruggeman model is better suited to predict the permeability. The Bruggeman equation was developed for the prediction of the dielectric constant of composite materials and has been adopted to simulate the gas transport properties of MMMs [45].

The Bruggeman model is described in Equation (13) below:

(13) $$\frac{P_{eff}}{P_{c}}=\frac{1}{{\left(1-\varphi_{d}\right)}^{3}}{\left(\frac{\frac{P_{eff}}{P_{c}}-\frac{P_{d}}{P_{c}}}{1-\frac{P_{d}}{P_{c}}}\right)}^{3}.$$

**Figure 10:**
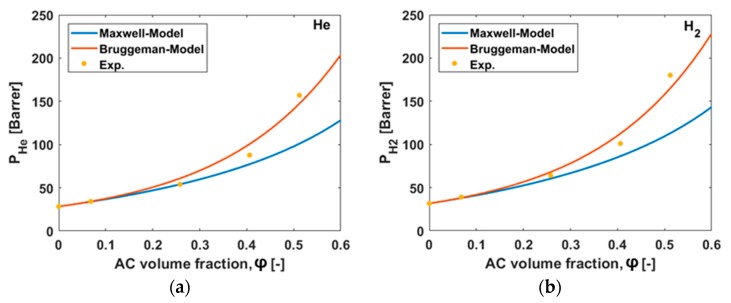

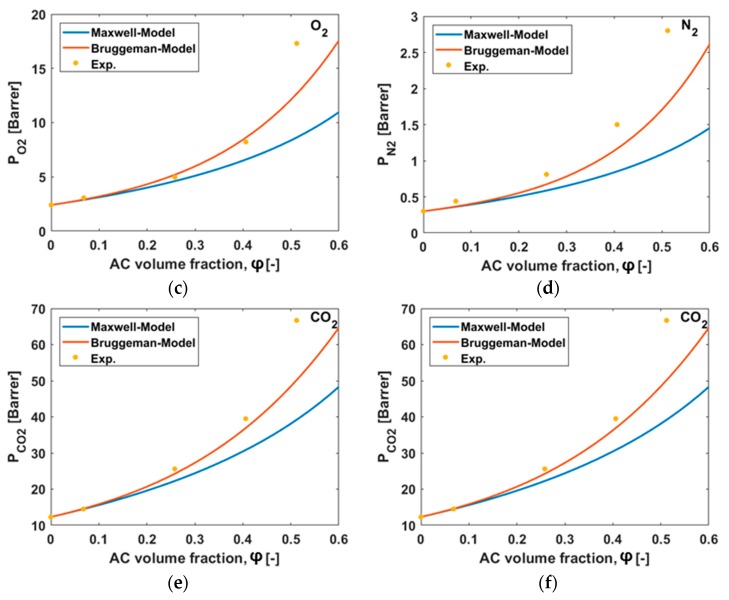
(**a**–**f**) Comparison of the experimental results (points) with the predictions of the Maxwell model (blue line) and the Bruggeman model (orange line) at 30 °C for the different gases and the different AC volume fraction used in this work.

In Figure 10, the model predictions for the gases He, H_2_, O_2_, N_2_, CO_2_, and CH_4_ compared to the experimental values are shown. Both models provide good predictions of the permeability for small AC volume fraction (0 < *φ_d_* < 0.2), while at a medium volume fraction (0 < *φ_d_* < 0.4) the Bruggeman model assumptions are still reasonable. At high AC volume fractions, the deviations for both models become more significant, but the trend reflects the experimental data. It should be mentioned that in the literature, many different approaches and models exist for the interpretation of the permeability of different gases in MMM [46,47,48,49], nevertheless for this work the simplest models of Maxwell and Bruggeman are used in order to avoid more assumptions. For example, the Lewis–Nielsen model can be applied, but the estimation of an accurate maximum packing volume fraction of the filler particles would be an additional assumption [50,51]. Most of the newer ideal models are enhancements of the Maxwell or Bruggeman models, and therefore the initial models were used to avoid additional assumptions.

Since both models predict the MMMs permeabilities in the valid regions for both models quite well, the hypotheses for predicting the permeability of AC is valid. By combining the results of the adsorption measurements for solubility with the results of the time-lag experiments, involving a contribution hypothesis for diffusivity, the results indicate that the overall MMM permeability is greatly influenced by the AC. That means that the experimental results for the permeability of the MMMs for the different gases are valid as well. 

## 4. Conclusions

Matrimid^®^ MMMs were prepared by introducing microporous AC particles up to a high volume content to investigate the effect of filler on the transport properties, thermal properties, and morphology. The obtained experimental results demonstrate that it is possible to form defect-free MMMs of Matrimid^®^ and AC. From the thermal analysis, the influence of AC content on the polymer was revealed. A small increase in the glass transition temperature was observed, indicating that the particles were well incorporated into the polymer matrix. The thermogravimetric analysis allowed for the estimation of the AC content with a better accuracy, which is important for the correlation of the results of the gas transport properties. The characterization by scanning electron microscopy indicated the good adaption of the AC in the polymer matrix, while the increase of AC content led to the partial concentration of the particles on the bottom side of the membranes without a decrease in the membrane stability, as it was also verified by the successful time-lag experiments. Both components are very well compatible when chloroform is used as a solvent and do not form any significant gaps on the AC particle and glassy polymer interface during the solvent evaporation even when the filler particles have very sharp edges and non-uniform shape. The filler particles have a significant influence on the gas transport properties of the studied membranes. The permeability coefficients of all gases increase with increasing the AC content while the selectivity remains stable for most of the gas pairs. The effect of the increasing permeabilities derives from the adsorption of the gases on the AC. With increasing content of AC in the MMMs, the influence on the sorption and diffusion, and finally the relative permeability increases. Hence, the results clearly demonstrate that the selected approach improves the membrane performance and allows for the fabrication of membrane materials closely approaching the upper bound identified by Robeson [3] for the investigated gas pairs. A prospect for further application is the preparation of MMMs with AC particles that incorporate into their porous structure substances, which are able to interact specifically with components of the gas mixture under separation. Hence, a further increase of the membrane performance appears to be possible.

## Figures and Tables

**Table 1:** Glass transition temperature of Matrimid^®^ in Matrimid^®^–AC MMMs.

Membrane—Activated Carbon Content	*T*_g_ [°C]
Matrimid^®^	319
Matrimid^®^ + 8 vol % AC	322
Matrimid^®^ + 31 vol % AC	324
Matrimid^®^ + 44 vol % AC	325
Matrimid^®^ + 50 vol % AC	326

**Table 2:** AC content of the MMMs according to the fabrication process and the TGA analysis. The volume content of AC included in the last column is calculated from the weight content determined from the TGA measurement.

*w_AC_^Theoretical^* [%]	*w_AC_^TGA^* [%]	$\varphi_{AC}$ [%]
4.70	6.00	8.20
20.00	23.94	30.50
33.33	36.32	44.20
42.85	42.11	50.30

**Table 3:** Permeability of the different MMMs with the different volume fraction of activated carbon *φ_AC_* at 30 °C.

$\varphi_{AC}$ [%]	P [Barrer] *					
	He	H_2_	O_2_	N_2_	CO_2_	CH_4_
**0**	28.3	31.6	2.41	0.30	12.3	0.34
**8**	34.2	39.0	3.06	0.44	14.5	0.43
**31**	53.8	63.8	4.97	0.81	25.6	0.67
**44**	87.8	101	8.22	1.50	39.5	1.06
**50**	157	180	17.3	2.80	66.7	2.25

* 1 Barrer = 10^−10^ [cm^3^_STP_/(cm s cmHg)] = 2.7 × 10^−9^ [Nm^3^/(m h bar)].

**Table 4:** Permselectivities for the gas pairs H_2_/CH_4_, CO_2_/CH_4_, and O_2_/N_2_ at 30 °C for the MMMs with different AC volume content and the corresponding Knudsen selectivity.

$\varphi_{AC}$ [%]	Permselectivity [-]		
	H_2_/CH_4_	CO_2_/CH_4_	O_2_/N_2_
**0**	94.4	36.8	8.11
**8**	90.2	33.4	7.04
**31**	95.7	38.3	6.16
**44**	95.8	37.4	5.48
**50**	80.2	29.7	6.19
**Knudsen selectivity**	2.82	0.60	0.94

**Table 5:** Solubility coefficient *S_eff_* of the different MMMs at 30 °C for the different AC volume content.

$\varphi_{AC}$ [%]	*S_eff_* [10^−3^ cm^3^_STP_/(cm^3^ cm Hg)]					
	He	H_2_	O_2_	N_2_	CO_2_	CH_4_
**0**	0.35	2.02	13.2	7.80	248	46.6
**8**	0.55	2.35	14.0	9.71	282	41.3
**31**	1.01	3.21	17.7	15.6	289	60.2
**44**	1.56	4.13	20.9	17.4	287	60.2
**50**	1.70	4.78	25.7	22.2	242	74.0

**Table 6:** Langmuir-isotherm equation parameters at 30 °C.

Gases	*q_max_* [mol/m^3^]	*b* [1/bar]
**He**	8069	0.0032
**H_2_**	7500	0.0067
**O_2_**	8270	0.0244
**N_2_**	5864	0.0357
**CO_2_**	10,479	0.1174
**CH_4_**	6788	0.0813

**Table 7:** Henry coefficient *H*, solubility coefficient *S*, and the ratio of them (*H*/*S*) for the studied gases at 30 °C.

Gases	*H* [mol/(m^3^·bar)]	*S* [mol/(m^3^·bar)]	*H*/*S* [-]
**He**	25.7	1.16	22.3
**H_2_**	50.3	6.76	7.43
**O_2_**	202	44.4	4.54
**N_2_**	209	26.2	7.99
**CO_2_**	1230	831	1.48
**CH_4_**	552	156	3.54

**Table 8:** Diffusion coefficients *D_eff_* of MMMs with different AC loading at 30°.

$\varphi_{AC}$ [%]	*D_eff_* [10^−7^ cm^2^/s]					
	He	H_2_	O_2_	N_2_	CO_2_	CH_4_
**0**	81.5	15.7	0.182	0.0381	0.0497	0.00719
**8**	62.0	16.6	0.219	0.0448	0.0512	0.0105
**31**	54.6	19.9	0.282	0.0518	0.0885	0.0111
**44**	56.4	24.5	0.393	0.0864	0.138	0.0176
**50**	92.9	37.7	0.673	0.126	0.275	0.0304

**Table 9:** Diffusion coefficient estimated by the time-lag measurements and permeability of the AC particles at 1000 mbar feed pressure as estimated by the diffusion and the Henry coefficients from the time-lag and sorption measurements, respectively.

Coefficients	He	H_2_	O_2_	N_2_	CO_2_	CH_4_
***D_AC_* [cm^2^/s]**	1.0 × 10^−5^	6.0 × 10^−6^	1.2 × 10^−7^	2.1 × 10^−8^	5.0 × 10^−8^	5.4 × 10^−9^
***P_AC_* [Barrer]**	800	898	70.2	13.4	184	8.84

## Nomenclature

*θ*	time-lag, [s]
*l*	membrane film thickness, [m]
*D_eff,i_*	effective diffusion coefficient of component *i*, [cm^2^/s]
*P_eff,i_*	effective permeability of component *i*, [Barrer]
*N_i_*	volumetric flowrate of component *i*, [Nm^3^/h]
*A_m_*	membrane area, [m^2^]
Δ*p_i_*	partial pressure difference between feed an permeate side of component *i*, [bar]
*S_eff,i_*	effective solubility coefficient of component *i*, [cm^3^_STP_/(cm^3^ cmHg)]; [mol/(m^3^ bar)]
*α_ij_*	selectivity of component *i* and *j*, [-]
*T*_g_	glass transition temperature, [°C]
*T*	temperature, [°C]
*w_AC_*	weight content of AC in MMM, [%]
*m_r,i_*	remaining relative mass of AC, MMM or Matrimid^®^, [%]
*φ_AC_*	volume content of AC in MMM, [%]
*ρ_i_*	density of component *i*, [kg/m^3^]
*M_i_*	molecular mass of the component *i*, [kg/mol]
*q*	adsorption concentration, [mol/m^3^]
*q_max_*	the maximum adsorbed concentration possible for a monolayer coverage, [mol/m^3^]
*p*	pressure, [bar]
*b*	parameter for an adsorbate-adsorbent pair, [1/bar]
*H*	Henry coefficient, [mol/(m^3^ bar)]
*c*	concentration, [mol/m^3^]
*P_c_*	permeability continuous phase, [Barrer]
*P_d_*	permeability dispersed phase, [Barrer]
*φ_d_*	volume fraction dispersed phase, [-]

## Subscripts

*eff*	effective
*AC*	activated carbon
*MMM*	mixed-matrix membrane
*g*	glass transition
*i*,*j*	gas component *i* or *j* (He, H_2_, O_2_, N_2_, CO_2_ or CH_4_)
*c*	continuous phase
*d*	dispersed phase
*Kn*	Knudsen
*r*	remaining relative mass
